# Supplementary material for: Characteristics of High-Resource Health System Users in Rural and Remote Regions: A Scoping Review
Source: Int J Environ Res Public Health. 2023 Apr 4;20(7):5385. doi: 10.3390/ijerph20075385 (PMC10094250; doi:10.3390/ijerph20075385)
Supplement: Supplementary file 1 [file ijerph-20-05385-s001.zip › ijerph-2244450-supplementary.pdf]

Table S1. Definition and limitations for search string.

Limitation	Definition
1. Search string	(“heavy use*” OR “frequent flyer” OR “heavy use*” OR “frequent attend*” OR “frequent consult*” OR “frequent use*” OR “high utiliz*” OR “high consult*” OR “high attend*” OR “high use*” OR “repeat use*” OR “recidivist*” OR “revolving door” OR “misuse” OR “hyperuse” OR “super use*”) AND ("health care" OR healthcare OR "health-care" OR "health service*" OR hospital OR clinic OR "emergency department" OR "emergency room") AND (rural* OR remote OR region*)
2. Time span	AND (PUBYEAR > 1999)
3. Language	AND (LIMIT-TO (LANGUAGE , "English")
4. Geographic	AND (LIMIT-TO (AFFILCOUNTRY , "Australia") OR LIMIT-TO (AFFILCOUNTRY , "Canada") OR (Canada OR Australia)
5. Type	AND (LIMIT-TO (DOCTYPE , "ar")

Table S2. Synthesis table of literature included in review.

Ref No	Author (Year)	Methods	Location	Sample	Target Population	Health Service(s) Analyzed	Relevant findings
[28]	Ansari et al. (2006)	Small area regression analysis	Victoria (Province), Australia	7,500	≥18 years old Living in private dwellings in Victoria	Ambulatory Care Sensitive Conditions	Lower income, education, rurality, and higher perceived barriers to care contributed to respondents' lower access ratings and higher ACSC hospitalization rates.
[35]	Ansari et al. (2013)	Retrospective statistical analysis	Victoria (Province), Australia	7,494	≥18 years old Living in private dwellings in Victoria	Ambulatory Care Sensitive Conditions	ACSC hospitalization rates associated with low-income, low education, smoking, difficulty accessing primary care, and lower quality outpatient service delivery.
[24]	Brameld et al. (2006)	Retrospective statistical analysis	Western Australia, Australia	10,082	Adults Completed the Western Australia 2000 Health and Wellbeing Survey	Hospital admissions	Hospitalization rates, average and total length of hospital stay, and readmission rates all increased with increasing distance that a patient lives from the hospital (level of remoteness).
[21]	Chen et al. (2021)	Retrospective statistical analysis	Ontario and Alberta, Canada	~2.7 million	≥18 years old At least 1 ED visit from April 1, 2015 – March 31, 2016	Emergency Department	High-system users were more likely to be female, older, of the lowest income quintile, live in rural area, have multiple comorbidities.
[31]	Chiu et al. (2022)	Retrospective statistical analysis	Québec, Canada	451,775	≥18 years old At least 1 ED visit from April 1, 2012 – March 31, 2013	Emergency Department	Individuals in these groups were more likely to be female, live in socially deprived and/or rural communities, and live in areas lacking community care services.
[16]	Dufour et al. (2020)	Retrospective cohort study	Québec, Canada	264,473	≥65 years old Living in Québec; consulted in an ED ≥1 time and diagnosed with ≥1 ACSC in the 2 years preceding	Ambulatory Care Sensitive Conditions	High-frequency use associated with older age, higher comorbidity index, common mental health disorders, higher material and social deprivation, and rural residence.
[26]	Garne et al. (2009)	Retrospective audit of the Royal Flying Doctor Service	Broken Hill, New South Wales, Australia (Remote)	9,395	All ages Accessed at least one RFDS service	Royal Flying Doctor Service	High-resource users had greater chronic health burdens.
[27]	Guilcher et al. (2016)	Retrospective cohort study	Ontario, Canada	587, 982	All ages Accounted for top 5% of health care expenditures	All health services	The high-cost health users were more likely to be ≥65 years old, female, from urban areas, and had a significant comorbidity burden.
[23]	Longman et al. (2012)	Cross-sectional survey	North Coast, New South Wales, Australia (Rural)	102	≥65 years old Residents of NSW with ≥3 admissions for ACS chronic conditions within 12-month period	Ambulatory Care Sensitive Conditions	People with significant comorbidity burdens (3 or more on CCI) were more likely to be very frequent users.
[29]	Manos et al. (2014)	Statistical analysis	Nova Scotia, Canada	326,555	12-24 years old All youth residents of Nova Scotia from 1997-2007	Youth care	Those in more rural locations and those in lower socioeconomic brackets utilized the health system in more expensive ways and with higher

Ref No	Author (Year)	Methods	Location	Sample	Target Population	Health Service(s) Analyzed	Relevant findings
							risk of being underserved in respect to their health needs.
[34]	Matsumuto et al. (2018)	Retrospective statistical analysis	Sioux Lookout, Ontario, Canada (Remote)	33,435	All ages<br>All ED users 2010-2014	Emergency department	High-frequency ED users accessed primary care services at more than twice the rate of non-high-frequency users and were more likely to present with benign presentations but frequently required admission.
[32]	Moe et al. (2021)	Retrospective statistical analysis	British Columbia, Canada	~1.2 million	≥18 years old<br>All ED users April 1, 2012 – March 31 2015	Emergency department	Frequent ER users were identified to be elderly, needing mental health care (all ages), and/or experiencing short term episodes of numerous visits.
[22]	Moe et al. (2022)	Retrospective statistical analysis	Ontario and Alberta, Canada	315, 975	≥18 years old<br>All ED users April 1, 2011 – March 31 2016	Emergency department	Frequent ED users had more comorbidities, were more likely to experience homelessness, live in rural communities, and be admitted with conditions of lower severity.
[25]	Palmer et al. (2014)	Retrospective database review	New Brunswick, Canada	59,803	All ages<br>All ED users in 12-month study period	Primary care	Predictors of frequent attendance included older age, being female, and having a listed PCP. High-resource use was more common in rural and remote regions.
[27]	Penning et al. (2016)	Retrospective cohort study	British Columbia, Canada (Province)	~1.1 million per year	≥50 years old<br>All BC residents registered to receive health services	All health services	Characteristics of high-resource GP, specialist, and hospital users include older adults, those in lower income quintiles, and poorer health status. Sex- and rurality-based trends changed depending upon services.
[36]	Quilty et al. (2016)	Unmatched case-control study	Northern Territory, Australia (Remote)	273	Adults<br>All Katherine ED users in 2012	Emergency department (non-chronic conditions)	Strong association between Aboriginal status, homelessness, and the involvement of alcohol in presentations.
[33]	Quilty et al. (2019)	Prospective cohort study	Northern Territory, Australia (Remote)	109	≥18 years old<br>Frequent attenders of Katherine ED identified by hospital staff	Emergency department	Frequent use associated with homelessness, overcrowded housing, food insecurity, low access to transport, alcohol misuse, and smoking.
[18]	Springer et al. (2017)	Retrospective cohort study	Northern Territory, Australia (Remote)	105,371	≥15 years old<br>All inpatient episodes in five NT hospitals from 2005-2014	Emergency department	Aboriginal patients accounted for a greater proportion of frequent users than non-Aboriginal patients. Aboriginal frequent users are generally younger and more likely to be female in comparison to non-Aboriginal patients.
[30]	Tiller er al. (2021)	Retrospective cohort study	Ontario and Alberta, Canada	199, 508	≥17 years old<br>Recipients of health care	Emergency department and hospital admissions – youth	For both inpatient hospital service and emergency department use, frequent use associated with being in the lower income quintile, facing more deprivation, were sicker, with more comorbidities, stay in the hospital longer, and require

Ref No	Author (Year)	Methods	Location	Sample	Target Population	Health Service(s) Analyzed	Relevant findings
							more intensive care when in hospital.
[19]	Vecchio et al. (2018)	Statistical Analysis	All provinces, Australia Nation-wide	80,000	≥20 years old Recipients of health care	Emergency department	Inadequate access to primary care and hospital services increased emergency department visits. Inadequate community care services significantly predicted emergency departments for people with physical, but not mental conditions.
[20]	Wallar et al. (2020)	Retrospective cohort study	All provinces (excluding Québec) Canada	389,065	18-74 years old (Excluded those living in Aboriginal communities, institutions, foster care, the province of Québec, and all military personnel)	Ambulatory Care Sensitive Conditions	Those who had an avoidable hospitalization tended to be older, rural, lower income, less educated, heavier smokers, never drinkers, obese, inactive, and have more chronic morbidities than those who had an unavoidable hospitalization or no hospitalization. Immigrant status was protective of high-resource use.
